# Morpho-Molecular Characterization Reveals a New Genus, Three Novel Species and Two New Host Records in Xylariomycetidae

**DOI:** 10.3390/jof10030189

**Published:** 2024-02-29

**Authors:** Wen-Li Li, Rui-Ru Liang, Jing Yang, Jian-Kui Liu

**Affiliations:** Center for Informational Biology, School of Life Science and Technology, University of Electronic Science and Technology of China, Chengdu 611731, China; wendy316@tom.com (W.-L.L.); lhh2022@tom.com (R.-R.L.); yangjing5633@gmail.com (J.Y.)

**Keywords:** four new taxa, *Amphisphaeria*, anthostomella-like, phylogeny, Xylariales

## Abstract

Xylariomycetidae comprises extremely diverse taxa that are widespread on decaying wood worldwide. An investigation of the diversity of microfungi on oil tree plantations in Sichuan Province was conducted during 2020–2021. Twelve saprobic taxa representing five species were identified as members of Amphisphaeriales and Xylariales through morphological comparisons. Phylogenetic analyses of combined ITS, LSU, *rpb2*, *tub2* and *tef1* sequence data indicated a distinct clade formed by three strains within Xylariomycetidae, unrelated to any currently recognized families. Thus, a novel anthostomella-like genus, *Bicellulospora*, is proposed and treated as Xylariales genera *incertae sedis*. *Bicellulospora* is characterized by dark brown to black, immersed, subglobose ascomata with a clypeus, cylindrical asci, and hyaline to yellowish brown, inequilaterally ellipsoidal ascospores with a large upper cell and a dwarf lower cell. Two new species of *Amphisphaeria*, namely *A. oleae* and *A. verniciae*, are introduced based on multi-gene phylogenetic analyses (ITS, LSU, *rpb2* and *tub2*) coupled with morphological characteristics. *Amphisphaeria micheliae* and *Endocalyx ptychospermatis* are reported as new host records.

## 1. Introduction

Xylariomycetidae is a phylogenetically and morphologically diverse fungal assemblage containing three orders, viz., Amphisphaeriales, Delonicicolales and Xylariales [1,2]. Many genera in the subclass are polyphyletic and paraphyletic, e.g., *Anthostomella*, *Biscogniauxia*, *Eutypa*, *Sporidesmium* and *Xylaria* [1,3,4,5,6,7]. Their morphologies have undergone convergent/divergent evolution but lack phylogenetic informativeness [8]. *Anthostomella* is a typical polyphyletic genus in Xylariales with highly variable morphological characteristics [1]. Species of *Anthostomella* are characterized by immersed ascomata, with or without a clypeus, amyloid or sometimes non-amyloid ascus apices, and brown ascospores bearing a gelatinous sheath or a hyaline dwarf cell [9]. Samarakoon et al. [1] divided *Anthostomella* into two clades, viz., clade “*A. formosa*” and clade “*A. helicofissa*”, based on multi-gene phylogenetic analyses and morphological features. Most species in the clade “*A. formosa*” have inequilaterally oblong–ellipsoidal ascospores with a hyaline dwarf cell, while species in the clade “*A. helicofissa*” have equilaterally ellipsoidal, dark brown, unicellular ascospores, with or without a mucilaginous sheath. *Anthostomella*-like species with a dwarf cell are accepted in different families as a polyphyletic character, e.g., *Entosordaria* (Barrmaeliaceae), *Occultitheca* (Xylariaceae), *Pyriformiascoma* (Xylariaceae) and *Vamsapriya* (Vamsapriyaceae) [1,10]. 

Cainiaceae was introduced by Krug [11] and was recently subsumed to Xylariales [1,12]. Based on the molecular data, *Endocalyx* was transferred from Apiosporaceae to Cainiaceae [13,14]. *Endocalyx* is a well-resolved genus in Cainiaceae, in which five species are accepted [14,15]. *Endocalyx* has a strong host specificity to palm but also occurs on dead vines, lilies or twigs of woody trees [16,17,18].

Amphisphaeriales was introduced by Eriksson and Hawksworth [19] and is phylogenetically a sister to Xylariales within Xylariomycetidae [20]. The family Amphisphaeriaceae was first introduced as “Amphisphaerieae” and was formally established as “Amphisphaeriaceae” by Winter [21]. Wijayawardene et al. [22] accepted *Amphisphaeria*, *Griphosphaerioma* and *Lepteutypa* in Amphisphaeriaceae, whereas *Lepteutypa* was synonymized to *Amphisphaeria* by Samarakoon et al. [23]. As a result of these studies, only *Amphisphaeria* and *Griphosphaerioma* are retained within Amphisphaeriaceae [1]. The sexual morph of *Amphisphaeria* has immersed, erumpent or rarely superficial, globose, subglobose or ellipsoidal ascomata; a two-layered peridium; cylindrical asci with a J+ or J− apical ring; and uniseriate, ellipsoidal to fusiform, 1–3-septate, hyaline or brown ascospores. The asexual morph is characterized by solitary or aggregated, globose, dark brown conidiomata; branched conidiophores; and hyaline, elongate–fusiform, one-celled conidia [20]. Members of *Amphisphaeria* are mostly discovered on dead plant materials in terrestrial and marine habitats [20,24,25,26,27].

During an investigation of fungal diversity on oil tree plantations in Sichuan Province from 2020 to 2021, a new anthostomella-like genus, *Bicellulospora*, was established and classified in Xylariales as genera *incertae sedis*, with *B. elaeidis* as the type species. Two new species of *Amphisphaeria*, namely *A. oleae* and *A. verniciae*, are introduced and justified by their morphological characteristics coupled with phylogenetic analyses. Two new host records, *Amphisphaeria micheliae* and *Endocalyx ptychospermatis*, are presented with detailed descriptions and illustrations. 

## 2. Materials and Methods

### 2.1. Sample Collection, Isolation and Morphology

Specimens were collected from decayed stems or twigs and were placed in paper bags or envelopes when taken to the laboratory. Macro–micro morphological observations were carried out, as mentioned in Samarakoon et al. [27]. Single-spore isolation was performed as described in Senanayake et al. [28]. Germinated spores were individually transferred to potato dextrose agar (PDA) plates and grown at 25 °C in the dark. Measurements were made with the Tarosoft (R) Image Framework program v. 0.9.7, following the procedures outlined by Liu et al. [8]. Photo plates representing fungal structures were processed in Adobe Photoshop CS6 software (Adobe Systems Inc., San Jose, CA, USA). 

Herbarium specimens were deposited in the herbarium of Cryptogams, Kunming Institute of Botany Academia Sinica (HKAS), Kunming, China, and the herbarium of the University of Electronic Science and Technology (HUEST), Chengdu, China. The isolates obtained in this study were deposited in China General Microbiological Culture Collection Center (CGMCC), Beijing, China, and the University of Electronic Science and Technology Culture Collection (UESTCC), Chengdu, China. MycoBank numbers were registered as outlined in MycoBank (http://www.MycoBank.org, accessed on 21 April 2023). 

### 2.2. DNA Extraction, PCR Amplification and Sequencing

Fresh mycelia scraped from 7-day-old isolates on PDA were used to extract genomic DNA using the EZ geneTM fungal gDNA kit (GD2416), according to the manufacturer’s instructions and protocols. The following loci were amplified and sequenced: the partial 28S large subunit rDNA (LSU), partial RNA polymerase II second largest subunit (*rpb2*), β-tubulin (*tub2*), translation elongation factor 1-alpha (*tef1*) and internal transcribed spacer (ITS). The forward and reverse primer pairs LR0R/LR5 [29], fRPB2-5F/fRPB2-7cR [30], T1/T22 [31], EF1-983F/EF1-2218R [32] and ITS5/ITS4 [33] were used to amplify the PCR fragments of these genes, respectively. The PCR amplification conditions followed those by Samarakoon et al. [23]. Sequencing of PCR products was performed by Beijing Tsingke Biological Engineering Technology and Sangon Biotech (Shanghai) Co., Ltd., Shanghai, China. 

### 2.3. Phylogenetic Analyses

The related sequences for phylogenetic analyses were downloaded from GenBank (Table S1). Single-gene sequences were aligned using the MAFFT v.7.429 online service (https://mafft.cbrc.jp/alignment/server/, accessed on 15 October 2022) [34], and the ambiguous sites were trimmed by TrimAI v1.2 [35]. The best-fitting evolutionary models for Bayesian analysis (BI) and maximum likelihood (ML) were determined independently for each gene by MrModeltest v. 2.3 [36]. Each sequence matrix was concatenated by the software SequenceMatrix v1.7.6 for multiple-gene phylogenetic analyses [37]. 

ML and BI were performed on the CIPRES Science Gateway platform [38]. ML analyses were made with RAxML-HPC2 on XSEDE v 8.2.8 with default parameters and bootstrapping with 1000 replicates [39]. MrBayes analyses were conducted in CIPRES with MrBayes on XSEDE 3.2.7a. Four simultaneous Markov chains were run for 20,000,000 generations, and trees were sampled every 1000th generation. The first 20% of the trees of the generations were discarded as burn-in, and the posterior probabilities (PP) were calculated from the remaining 80% of the trees [40]. BI posterior probabilities and maximum likelihood bootstrap values equal to or greater than 0.95/75% are indicated near each node of the phylogenetic tree. 

Phylograms were visualized on FigTree v.1.4.0 [41], and the layouts of the trees were drawn in the Adobe Illustrator CS6 software (Adobe Systems, USA). All newly generated sequences in the study were deposited in GenBank, and relevant sequences for phylogenetic analyses are included in the Table S1.

## 3. Results

### 3.1. Phylogenetic Analyses

Two phylogenetic analyses were conducted to resolve the relationships among taxa in *Amphisphaeria* (Amphisphaeriaceae) and Xylariomycetidae. 

**Figure 1:**
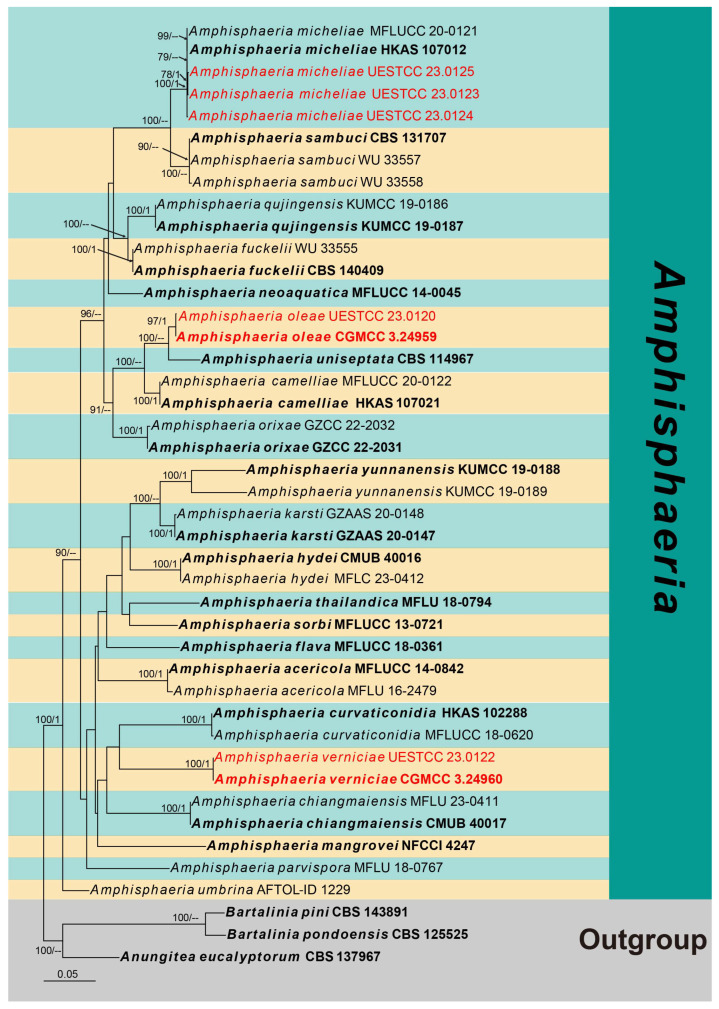
Phylogram generated from RAxML analysis based on the combined ITS, LSU, *rpb2* and *tub2* sequence data of *Amphisphaeria* isolates. Bootstrap values for maximum likelihood of ≥75% and Bayesian posterior probabilities of ≥0.95 are given near the nodes as ML/PP. Isolates from this study are marked in red, and ex-type strains are in bold.

The first phylogenetic tree represents the genus *Amphisphaeria* (Figure 1). Seven strains obtained in this study were analyzed with other taxa of *Amphisphaeria* based on the concatenated dataset of ITS, LSU, *rpb2* and *tub2*. The data matrix comprised 43 taxa, with *Anungitea eucalyptorum* (CBS 137967), *Bartalinia pini* (CBS 143891) and *B. pondoensis* (CBS 125525) as the outgroup taxa. The dataset consists of 3333 total characters (ITS: 1–648 bp, LSU: 649–1483 bp, *rpb2*: 1484–2462 bp, *tub2*: 2463–3333 bp, including gaps). These seven strains clustered within *Amphisphaeria*, representing two new and one known species. Two strains of *A. verniciae* (CGMCC 3.24960 and UESTCC 23.0122) formed a distinct clade as a sister to *A. curvaticonidia*, while two strains of *A. oleae* (CGMCC 3.24959 and UESTCC 23.0120) were sisters to *A. uniseptata* (CBS 114967) with 100% ML statistical support. The remaining three strains (UESTCC 23.0123, UESTCC 23.0124 and UESTCC 23.0125) grouped with *A. micheliae*, forming a well-supported clade (100% ML/1.00 PP). 

**Figure 2:**
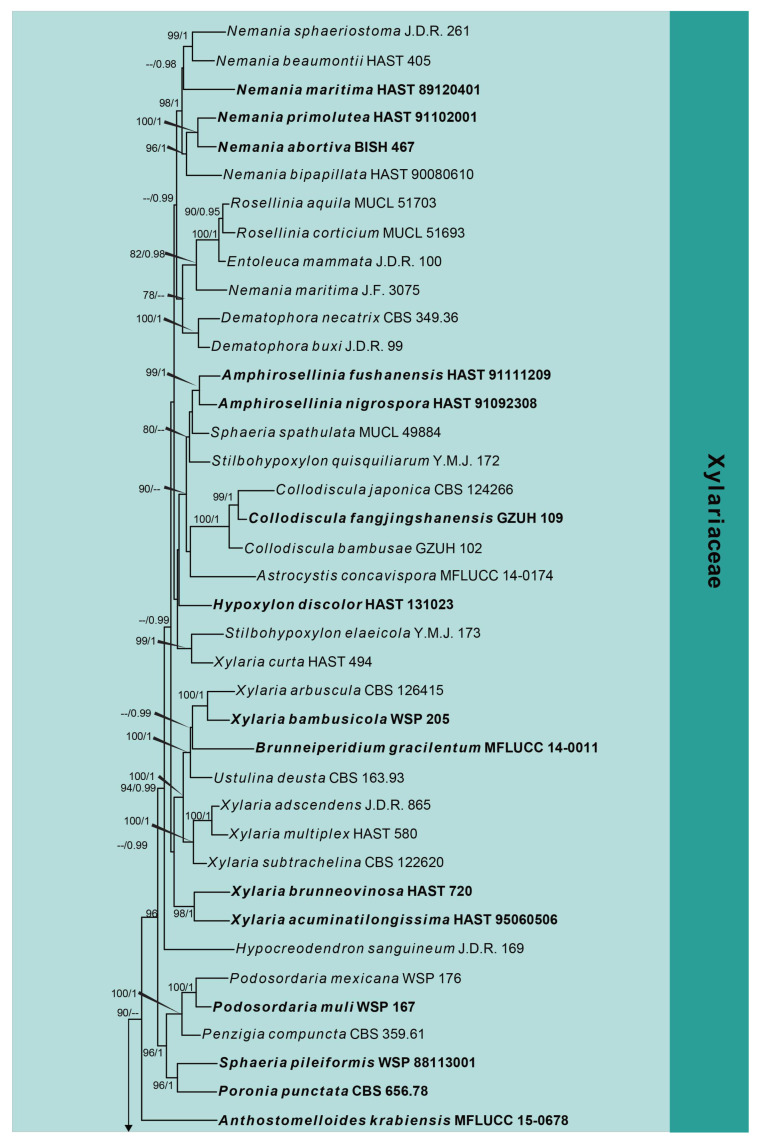

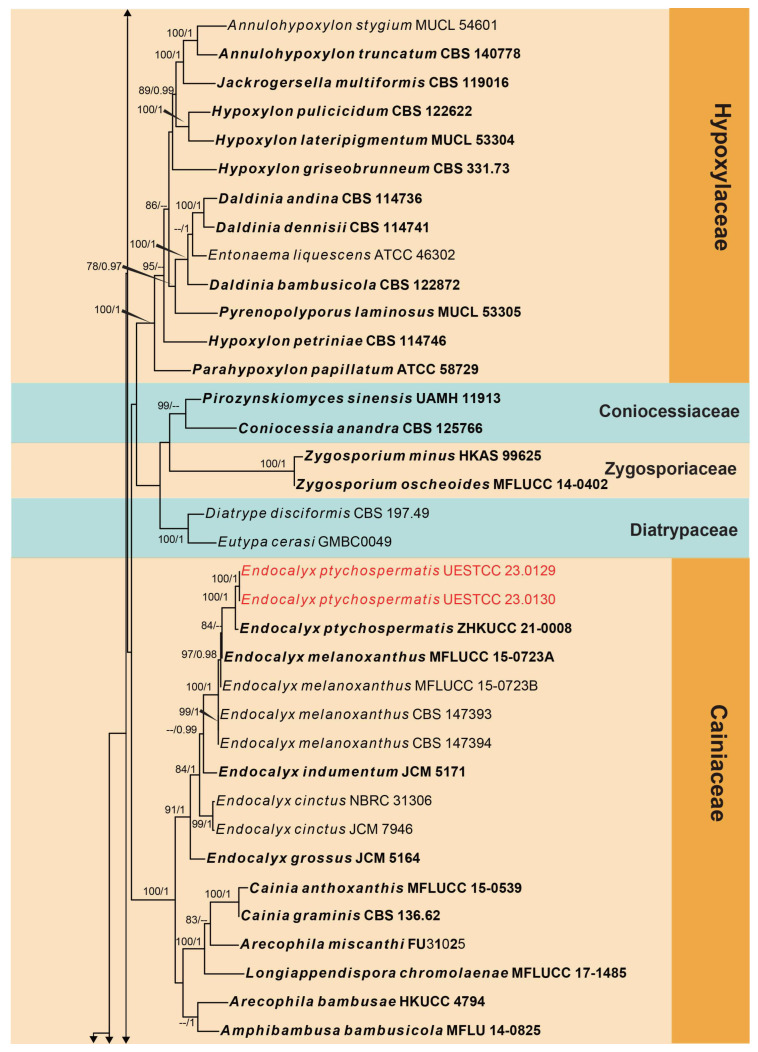

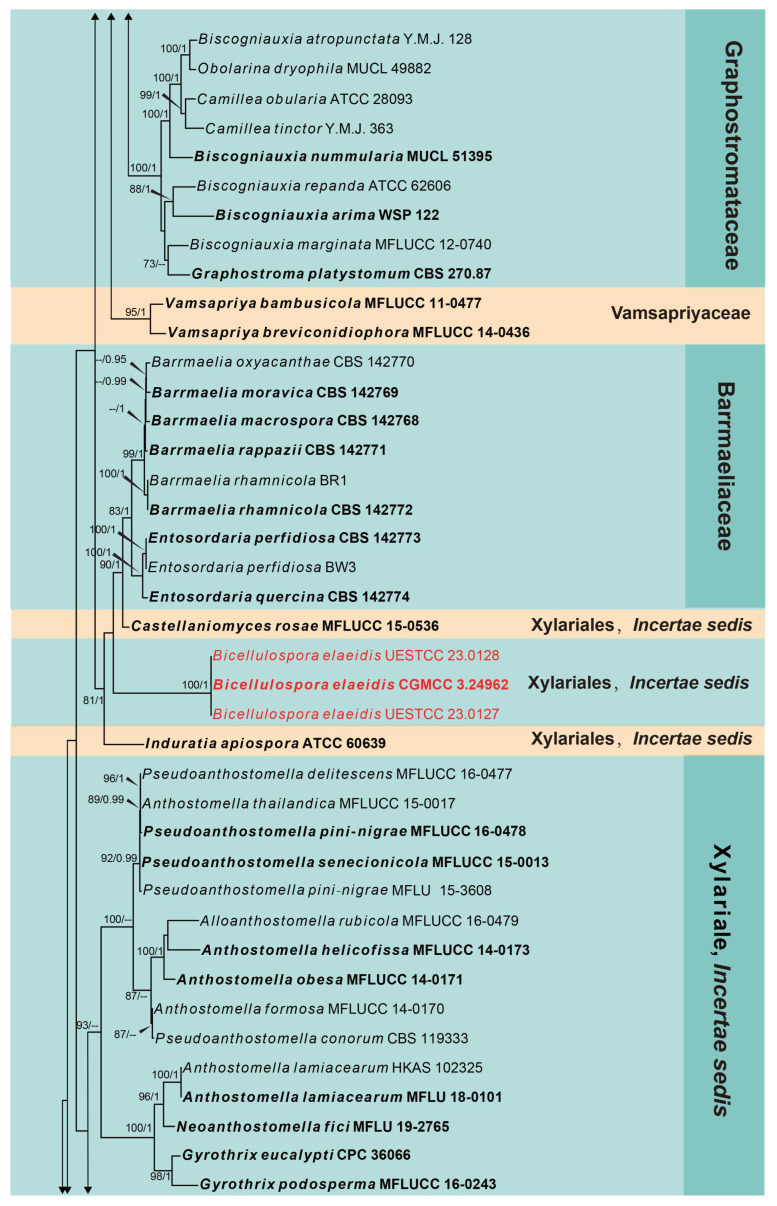

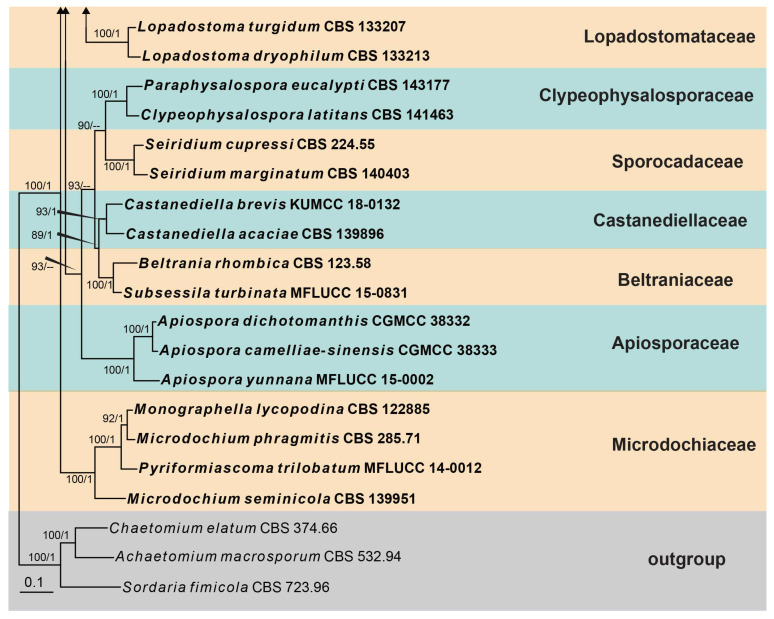
Phylogram generated from RAxML analysis based on the combined ITS, LSU, *rpb2*, *tub2* and *tef1* sequence matrix of Xylariomycetidae. Bootstrap values for maximum likelihood of ≥75% and Bayesian posterior probabilities of ≥0.95 are given near the nodes as ML/PP. Isolates obtained from this study are in red, and ex-type strains are in bold.

The second phylogeny represents the subclass Xylariomycetidae (Figure 2). Five strains obtained in this study were analyzed with other taxa of Xylariomycetidae based on the concatenated dataset of ITS, LSU, *rpb2*, *tub2* and *tef1*. The data matrix comprised 135 taxa, with *Achaetomium macrosporum* (CBS 532.94), *Chaetomium elatum* (CBS 374.66) and *Sordaria fimicola* (CBS 723.96) as the outgroup taxa. The dataset consists of 4849 total characters (ITS: 1–478 bp, LSU 479–1827 bp, *rpb2* 1828–3021 bp, *tub2* 3022–4141 bp, *tef1*: 4142–4849 bp, including gaps). Three strains (CGMCC 3.24962, UESTCC 23.0127 and UESTCC 23.0128) formed a separate clade in Xylariales close to Barrmaeliaceae and *Castellaniomyces rosae* (MFLUCC 15-0536), with poor statistical support. The other two strains (UESTCC 23.0129 and UESTCC 23.0130) clustered with *Endocalyx ptychospermatis* (ZHKUCC 21-0008), with absolute bootstrap support (100% ML/1.00 PP).

### 3.2. Taxonomy 

**Figure 3:**
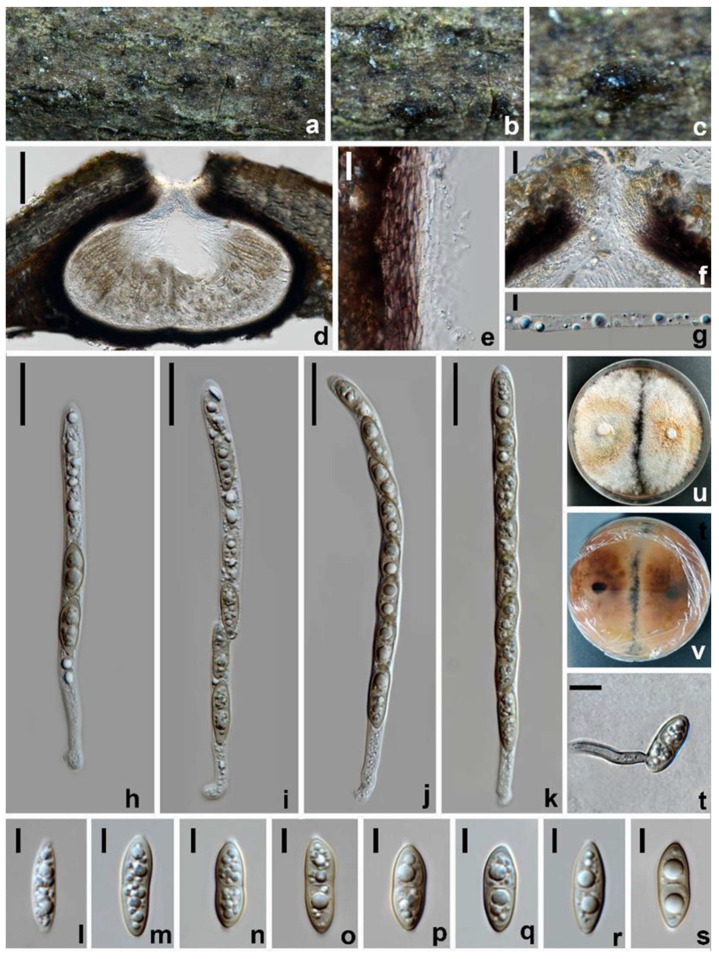
*Amphisphaeria micheliae* (HUEST 23.0123, new host record). (**a**–**c**) The appearance of ascomata on substrate. (**d**) Vertical section of an ascoma. (**e**) Peridium. (**f**) Ostiole. (**g**) Paraphyses. (**h**–**k**) Asci. (**l**–**s**) Ascospores. (**t**) Germinated ascospore. (**u**) Colonies on PDA from above. (**v**) Colonies on PDA from below. Scale bars: (**d**) 100 µm; (**e**,**g**,**t**) 10 µm; (**f**) 40 µm; (**h**–**k**) 20 µm; (**l**–**s**) 5 µm.

*Amphisphaeria micheliae* Samarak., Jian K. Liu & K.D. Hyde, *Journal of Fungi* 6(3): 16 (2020) (Figure 3).

MycoBank number: MB 836112.

*Saprobic* on dead branches of *Acer truncatum*. Sexual morph: *Ascomata* 200–270 × 380–505 µm ($\bar{x}$ = 240 × 470 µm, *n* = 10), dark brown to black, immersed, solitary, scattered, subglobose or oblate. *Ostiole* 56–67 µm wide, centric. *Peridium* 20–40 µm wide, leathery, two-layered; outer layer consisting of reddish brown, polygonal to elongated, thick-walled cells of *textura angularis*; inner layer with hyaline, flattened, thin-walled cells of *textura angularis. Paraphyses* 3–5.5 µm wide, hyaline, cellular, septate, guttulate. *Asci* 96–147 × 6.5–9.5 µm ($\bar{x}$ = 118 × 8.5 µm, *n* = 30), eight-spored, cylindrical, with an elongated pedicel and a discoid apical ring. *Ascospores* 17–22 × 6.5–7.5 µm ($\bar{x}$ = 19 × 7 µm, *n* = 30), uniseriate, partially overlapping, narrowly ellipsoidal to fusiform, one-septate, becoming slightly narrower towards both ends, initially hyaline, pale yellow to yellowish brown when aged, guttulate. Asexual morph: Undetermined.

Culture characteristics: Colonies on PDA reaching 13–17 mm diam. after one week at 25 °C in the dark, colonies circular, flattened, dense, with a rough surface, concentrically zonate, milky white to pale reddish brown, mycelium velvety; from below: reddish brown to dark brown at the center, pale brown at the margin, producing yellow pigments in PDA.

Materials examined: China, Sichuan Province, Chengdu city, Pidu district, 30°49′26.76″ N, 103°47′41.24″ E, elevation 442 m, on branches of *Acer truncatum*, 19 March 2021, W.L. Li, 095 (HUEST 23.0123), living culture UESTCC 23.0123; *ibid*., Leshan city, Jingyan county, 29°30′23.23″ N, 103°57′30.52″ E, elevation 410 m, on branches of *Idesia polycarpa*, 23 July 2021, W.L. Li, 319 (HUEST 23.0124), living culture UESTCC 23.0124; *ibid*., Mianyang city, Youxian district, 31°22′28.88″ N, 104°50′42.00″ E, elevation 398 m, on branches of *Olea europaea*, 10 June 2021, W.L. Li, 277 (HUEST 23.0125), living culture UESTCC 23.0125.

Notes: The multi-gene phylogenetic analyses showed our isolates (UESTCC 23.0123, UESTCC 23.0124 and UESTCC 23.0125) to be clustered with *Amphisphaeria micheliae*, with 100% ML/1.00 PP statistical support (Figure 1). Morphologically, the new collections share similar characteristics with *A. micheliae* (MFLUCC 20-0120 and HKAS 107012). However, the colony of the strain UESTCC 23.0123 produces yellowish brown pigments on PDA, while pigmentation is not observed in the ex-type strain MFLUCC 20-0121 in the same medium [23]. Considering that light, temperature, humidity and the incubation time can affect the colony morphology, the new collections are identified as *A. micheliae*.

**Figure 4:**
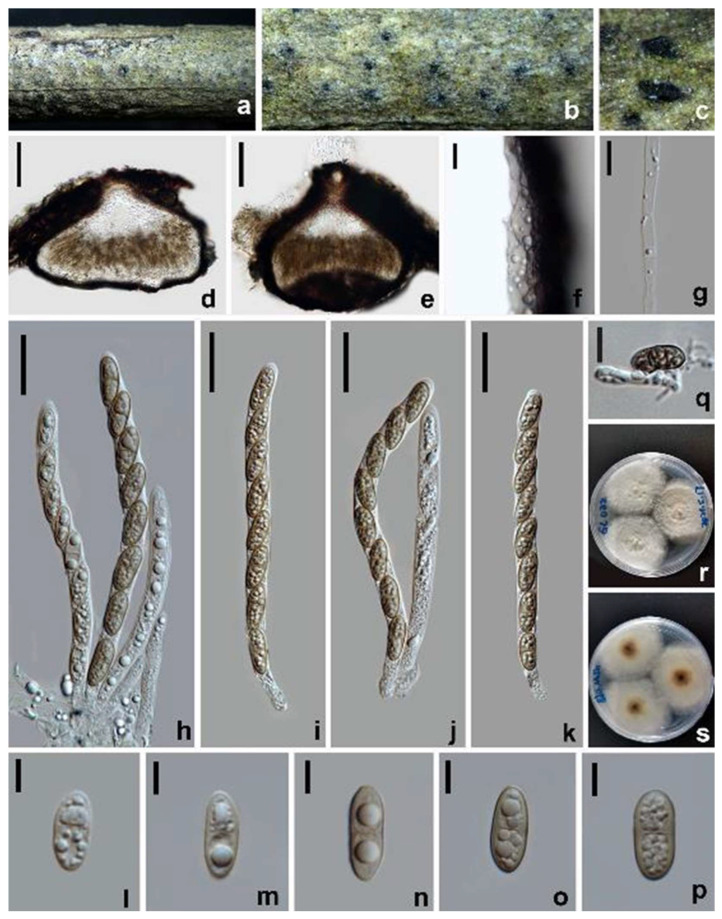
*Amphisphaeria oleae* (HKAS 128843, holotype). (**a**–**c**) The appearance of ascomata on substrate. (**d**,**e**) Vertical section of ascomata. (**f**) Peridium. (**g**) Paraphyses. (**h**–**k**) Asci. (**l**–**p**) Ascospores. (**q**) Germinated ascospore. (**r**) Colonies on PDA from above. (**s**) Colonies on PDA from below. Scale bars: (**d**,**e**) 100 µm; (**f**,**g**,**q**) 10 µm; (**h**–**k**) 20 µm; (**l**–**p**) 5 µm.

*Amphisphaeria oleae* W.L. Li, R.R. Liang & Jian K. Liu, sp. nov. (Figure 4).

MycoBank number: 849633.

Etymology: The epithet ‘oleae’ refers to the host genus *Olea*, on which the fungus was collected.

Holotype: HKAS 128843.

*Saprobic* on decaying branches of *Olea europaea*. Sexual morph: *Ascomata* 210–240 × 290–375 µm ($\bar{x}$ = 230 × 320 µm, *n* = 10), immersed, visible as black spots, surrounded by a pale gray halo on the surface, solitary, aggregated, globose to subglobose, ostiolate, papillate. *Ostiole* 55–87 µm diam., periphysate, conical or circular. *Peridium* 23.5−32.5 µm thick, consisting of multi-layered cells of *textura angularis*; inner layers with hyaline to pale gray, thin-walled cells; outer layers with dark brown to black, thick-walled cells. *Paraphyses* 3.5−5 µm wide, hyaline, filamentous, septate, guttulate. *Asci* 91–115 × 7–8.5 µm ($\bar{x}$ = 101 × 7.5 µm, *n* = 30), eight-spored, unitunicate, cylindrical, with a cylindrical pedicel and an apical ring. *Ascospores* 13–15 × 4.5–7 µm ($\bar{x}$ = 14 × 6 µm, *n* = 30), L/W 2.2, uniseriate, ellipsoidal, hyaline when young, becoming yellowish brown at maturity, with a median septum, smooth- and thick-walled, guttulate. Asexual morph: Undetermined.

Culture characteristics: Ascospores germinating on PDA within 12 h at 25 °C. Colonies growing on PDA reaching 3.2–3.4 cm diam. after one week at 25 °C in the dark, white to pale yellow, circular, flat, center denser than the edge; in reverse, white becoming yellowish brown to brown from the center with age.

Materials examined: China, Sichuan Province, Guangyuan city, Chaotian district, 30°19′57.04″ N, 103°59′46.66″ E, elevation 432 m, on branches of *Olea europaea*, 30 January 2021, W.L. Li, 32a (HKAS 128843, holotype), ex-type living culture CGMCC 3.24959; 32b (HUEST 23.0120, isotype), ex-isotype living culture UESTCC 23.0120. 

Notes: The multi-gene phylogeny indicated that *Amphisphaeria oleae* (CGMCC 3.24959) is nested within *Amphisphaeria* and clustered with *A. uniseptata* (CBS 114967) in a sister clade with 100% ML statistical support (Figure 1). The BLASTn search of the *tub2* sequence of *A. oleae* (CGMCC 3.24959) showed 92.61% (351/379 bp, eight gaps) similarity with *A. camelliae* (MFLU 20-0181). Comparisons of the *tub2* sequence of *A. oleae* (CGMCC 3.24959) and *A. uniseptata* (CBS 114967) showed 85% sequence identity (257/301 base pairs (bp), 12 gaps). Morphologically, *A. oleae* resembles *A. uniseptata* in having cylindrical asci and uniseriate, ellipsoidal, hyaline to brown ascospores with a median septum, but differs from the latter by having a two-layered ascomatal wall (Figure 4e) (vs. three-layered) and smaller ascospores (L/W 2.4 vs. L/W 2.7) [42]. Thus, *A. oleae* is hereby introduced as a novel species in *Amphisphaeria*. 

**Figure 5:**
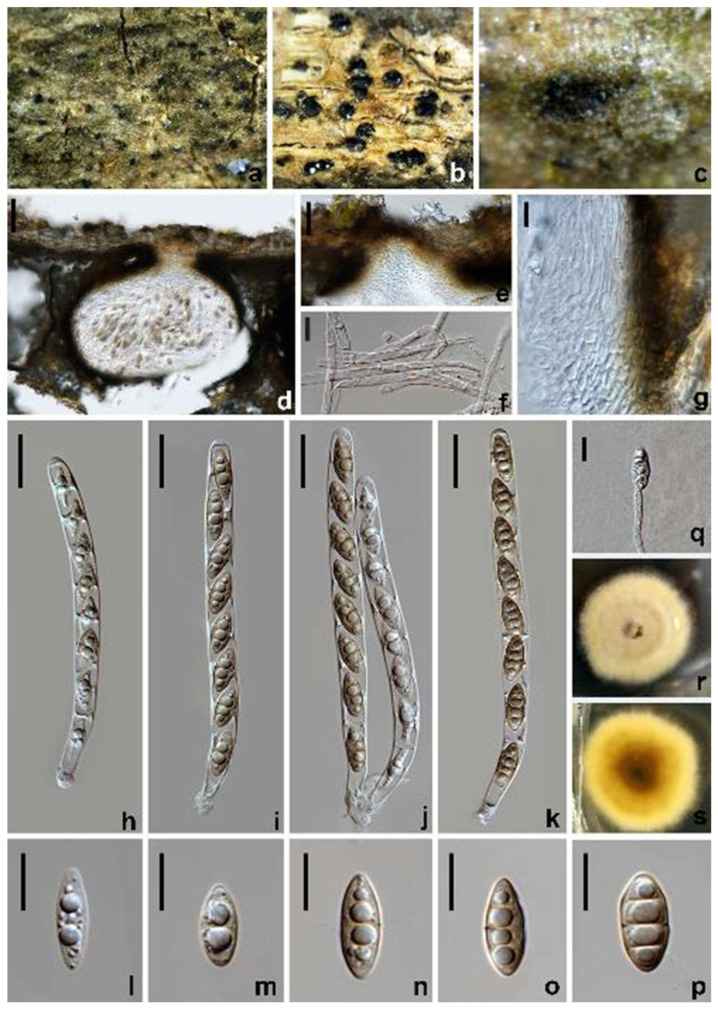
*Amphisphaeria verniciae* (HKAS 128844, holotype). (**a**–**c**) The appearance of ascomata on substrate. (**d**) Vertical section of an ascoma. (**e**) Ostiole. (**f**) Paraphyses. (**g**) Peridium. (**h**–**k**) Asci. (**l**–**p**) Ascospores. (**q**) Germinated ascospore. (**r**) Colony on PDA from above. (**s**) Colony on PDA from below. Scale bars: (**d**) 50 µm; (**e**) 40 µm; (**f**,**g**,**l**–**q**) 10 µm; (**h**–**k**) 20 µm.

*Amphisphaeria verniciae* W.L. Li, R.R. Liang & Jian K. Liu, sp. nov. (Figure 5).

MycoBank number: 849634.

Etymology: The epithet ‘verniciae’ refers to the host genus *Vernicia*, on which the holotype was collected.

Holotype: HKAS 128844.

*Saprobic* on decaying branches of *Vernicia fordii*. Sexual morph: *Ascomata* 110–200 × 130–240 µm ($\bar{x}$ = 150 × 170 µm, *n* = 10), immersed, visible as black spots, globose to subglobose, solitary, scattered, ostiolate, papillate. *Ostiole* 37–53 µm diam., periphysate, conical or circular. *Peridium* 23−30.5 µm thick, consisting of multi-layered cells of *textura angularis*; inner layers with hyaline, polyhedral to elongated cells; outer layers with dark brown to brown cells. *Paraphyses* 2.5−5 µm wide, hyaline, filamentous, septate. *Asci* 111–143 × 9–13.5 µm ($\bar{x}$ = 130 × 11 µm, *n* = 30), eight-spored, unitunicate, cylindrical, with a short pedicel and a minute thin apical ring. *Ascospores* 16–19 × 6–7.5 µm ($\bar{x}$ = 17 × 6.5 µm, *n* = 30), L/W 2.4, uniseriate, narrowly fusiform to ellipsoidal, hyaline, bi-guttulate when immature, becoming pale brown, multi-guttulate when aged, 0–3-septate. Asexual morph: Undetermined.

Culture characteristics: Ascospores germinating on PDA within 12 h at 25 °C. Colonies growing on PDA reaching 2–2.3 cm diam. after one week at 25 °C in the dark, white to pale yellow, circular, flat, with a smooth surface and entire margin; in reverse, yellowish brown at the center, paler towards the margin.

Materials examined: China, Sichuan Province, Guangyuan city, Chaotian district, 32°41′05.48″ N, 106°01′22.40″ E, elevation 628 m, on dead branches of *Vernicia fordii*, 19 April 2021, W.L. Li, 217a (HKAS 128844, holotype), ex-type living culture CGMCC 3.24960; 217b (HUEST 23.0121, isotype), ex-isotype living culture UESTCC 23.0121. 

Notes: The BLASTn searches of the LSU sequence of *Amphisphaeria verniciae* (CGMCC 3.24960) resulted in 97.62% (861/882 bp, 1 gap) and 97.38% (854/877 bp, 1 gap) matches with *A. fuckelii* (LEF1) and *A. camelliae* (HKAS 107021). The *rpb2* and *tub2* BLASTn results showed 82.65% (872/1055 bp, two gaps) and 90.34% (870/963 bp, nine gaps) matches with *A. fuckelii* (LEF1), respectively. The multi-gene phylogeny (Figure 1) showed that *A. verniciae* (CGMCC 3.24960 and UESTCC 23.0122) is closely related to *A. curvaticonidia*. Morphologically, *A. verniciae* is comparable to *A. camelliae*, *A. curvaticonidia* and *A. fuckelii*. However, *A. curvaticonidia* and *A. fuckelii* can be distinguished from *A. verniciae* by their cylindrical ascospores with rounded ends [43]. Ascospores of *A. verniciae* are mostly three-septate, while in *A. camelliae*, they are often one-septate [23,43]. In addition, they are different in terms of the L/W of ascospores (*A. camelliae* L/W 3.1 vs. *A. curvaticonidia* L/W 2.7, *A. fuckelii* L/W 2.9 vs. *A. verniciae* L/W 2.4) [23,43]. Therefore, we introduce *A. verniciae* as a new species in *Amphisphaeria*.

*Bicellulospora* W. L. Li, R. R. Liang & Jian K. Liu, gen. nov.

MycoBank number: 849635.

Etymology: ‘*Bicellulospora’* refers to the two-celled ascospores.

*Saprobic* on wood or bark. Sexual morph: *Ascomata* immersed to semi-immersed, black, globose to subglobose, solitary or in small groups, ostiolate. *Ostiole* pore rounded, centric. *Peridium* multi-layered, comprising pale brown to dark brown cells of *textura angularis. Paraphyses* numerous, filamentous, hyaline, septate, unbranched, guttulate, smooth. *Asci* eight-spored, unitunicate, cylindrical, with a short stipe, apically rounded, with a discoid apical ring. *Ascospores* uniseriate or partially overlapping, inequilaterally oblong–ellipsoidal, with a lower deeply constricted septum, hyaline when young, becoming olivaceous brown at maturity, smooth-walled, lacking a germ slit and a sheath. Asexual morph: Undetermined. 

Type species—*Bicellulospora elaeidis.*

Notes: The phylogenetic analyses revealed that three new collections (CGMCC 3.24962, UESTCC 23.0127 and UESTCC 23.0128) formed a distinct lineage in Xylariales. They clustered with Barrmaeliaceae (*Barrmaelia* and *Entosordaria*) and *Castellaniomyces rosae* (MFLUCC 15-0536), with poor statistical support. Species of *Barrmaelia* can be separated from *Bicellulospora* by their one-celled ascospores without a dwarf cell. *Entosordaria* possesses ellipsoid to allantoid, two-celled ascospores consisting of a dark brown larger cell and a small, hyaline dwarf cell [10]. *Castellaniomyces rosae* differs from *Bicellulospora* in having elongated fusiform, brown to dark brown, and equally two-celled ascospores surrounded by a thick mucilaginous sheath [44]. 

*Bicellulospora* is similar to *Anthostomella*, *Apiospora*, *Occultitheca*, *Pyriformiascoma* and *Vamsapriya* in having inequilaterally ellipsoidal ascospores consisting of a larger cell and a dwarf cell [1,10,44,45,46,47,48]. However, *Occultitheca* (*O. rosae*) can be easily distinguished from *Bicellulospora* by a basal hyaline dwarf cell and a well-visible germ slit on the ascospores [1]. *Apiospora* (*A. guiyangensis*) and *Vamsapriya* (*V. mucosa*) differ from *Bicellulospora* in having ellipsoid to reniform, hyaline ascospores [2]. *Pyriformiascoma* (*P. trilobatum*) differs from *Bicellulospora* in possessing two-celled ascospores with a brown or olivaceous green larger cell and a hyaline dwarf cell. Additionally, its asci with cytoplasm form a fork-like invagination below the indistinct apical apparatus [45]. Although several *Anthostomella* species (e.g., *A. castnopsis*, *A. clypeata*, *A. cynaroides*, *A. formosa*, *A. proteae* and *A. tomicoides*) have similar ascus and ascospore shapes to *Bicellulospora elaeidis*, they can be distinguished through detailed comparisons (Table 1). Based on the phylogenetic analyses and morphological characteristics, the new genus *Bicellulospora* is established in Xylariales as genera *incertae sedis*, with *B. elaeidis* designed as the type species.

**Figure 6:**
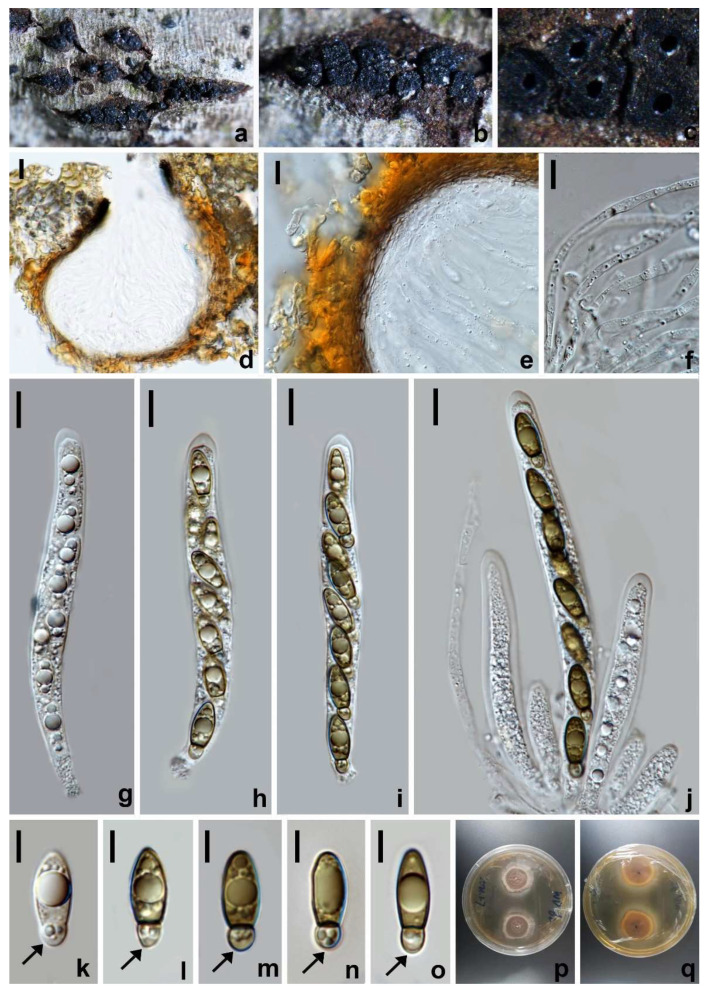
*Bicellulospora elaeidis* (HKAS 128845, holotype). (**a**–**c**) The appearance of ascomata on substrate. (**d**) Vertical section of an ascoma. (**e**) Peridium. (**f**) Paraphyses. (**g**–**j**) Asci. (**k**–**o**) Ascospores; the structures indicated by the arrows are dwarf cells. (**p**) Colonies on PDA from above. (**q**) Colonies on PDA from below. Scale bars: (**d**) 20 µm; (**e**,**f**,**g**–**j**) 10 µm; (**k**–**o**) 5 µm.

*Bicellulospora elaeidis* W.L. Li, R.R. Liang and Jian K. Liu, sp. nov. (Figure 6).

MycoBank number: 849636.

Etymology: The epithet ‘elaeidis’ refers to the host genus *Elaeis*, on which the holotype was collected.

Holotype: HKAS 128845.

*Saprobic* on decaying branches of *Elaeis guineensis*. Sexual morph: *Ascomata* 140–170 × 120–140 µm ($\bar{x}$ = 160 × 130 µm, *n* = 10), semi-immersed, scattered or gregarious, globose to subglobose, uniloculate, black, papillate, with a central ostiole. *Ostiole* 55–87 µm diam., central. *Peridium* 14−24 µm thick, coriaceous, thin-walled, inner layer of hyaline cells of *textura angularis*, outer layer of pale brown to dark brown cells of *textura angularis*. *Paraphyses* 3−4.5 µm wide, hyaline, septate, guttulate. *Asci* 85.5–100 × 7.5–10 µm ($\bar{x}$ = 94 × 9 µm, *n* = 30), eight-spored, unitunicate, cylindrical, with a short stipe, apically rounded, with a flattened apical ring. *Ascospores* 14.5–17.5 × 5.5–6.5 µm ($\bar{x}$ = 16 × 6 µm, *n* = 30), uniseriate or partially overlapping, inequilaterally oblong–ellipsoidal, hyaline when young, becoming olivaceous brown when mature, smooth-walled, with a lower constricted septum at about one-quarter of the length to the base; larger cells bullet-shaped, containing a big guttule and several small guttules; lacking a germ slit and a sheath, dwarf cells subglobose, containing 2–3 small guttules, sometimes slightly paler than the larger cells. Asexual morph: Undetermined.

Culture characteristics: Ascospores germinating on PDA within 48 h at 25 °C. Colonies growing on PDA reaching 1.9−2.2 cm diam. after two weeks at 25 °C in the dark, circular, velvety, pinkish brown in the middle, white at the outer rings, with dense mycelia in the inner rings, sparse at the entire margin; in reverse, brown in the center, pale yellow at the middle ring and white at the margin. 

Materials examined: China, Sichuan Province, Chengdu city, University of Electronic Science and Technology of China (UESTC), 30°45′24.71″ N, 103°55′21.07″ E, elevation 465 m, on dead branches of *Elaeis guineensis*, 30 November 2020, W.L. Li, W62a (HKAS 128845, holotype), ex-type living culture CGMCC 3.24962; W62b (HUEST 23.0127, isotype), ex-isotype living culture UESTCC 23.0127; W62c (HUEST 23.0128, isotype), isotype living culture UESTCC 23.0128.

Notes: *Bicellulospora elaeidis* was identified as a new species isolated from the dead branches of *Elaeis guineensis*. The BLASTn search of the LSU sequence of *Bi. elaeidis* (CGMCC 3.24962) showed 97.07% (893/920 bp, 8 gaps) and 94.94% (939/989 bp, 24 gaps) similarity with *Entosordaria perfidiosa* (BW3) and *Barrmaelia rhamnicola* (BR), respectively. The BLASTn search of the *tef1* sequences resulted in 87.81% (850/968 bp, seven gaps) and 87.51% (848/969 bp, nine gaps) similarity with *E. perfidiosa* (EPE) and *Ba. rappazii* (Cr2), respectively. The ITS sequence of *Bi. elaeidis* (CGMCC 3.24962) presented only 86.07% (488/567 bp, 25 gaps) and 85.69% (491/573 bp, 26 gaps) similarity with *Ba. oxyacanthae* (BO) and *E. quercina* (CBS 142774), respectively. The multi-gene analyses indicated that *Bi. elaeidis* (CGMCC 3.24962, UESTCC 23.0127 and UESTCC 23.0128) formed a distinct clade that is a sister to the clade containing *Barrmaelia*, *Castellaniomyces* and *Entosordaria* (Figure 2). However, they have significant differences in morphology.

**Figure 7:**
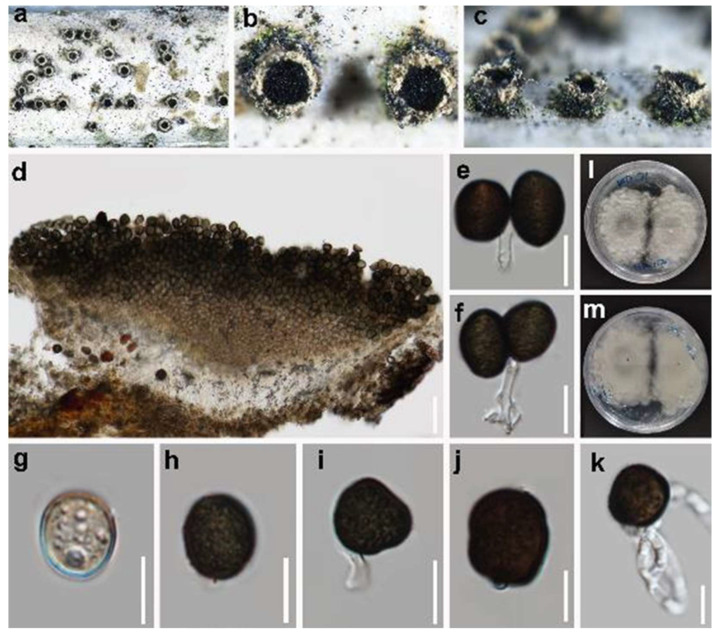
*Endocalyx ptychospermatis* (HUEST 23.0129). (**a**–**c**) The appearance of conidiomata on the substrate. (**d**) Vertical section of a conidioma. (**e**,**f**) Conidiphore and conidia. (**g**–**j**) Conidia. (**k**) Germinated conidium. (**l**) Colonies on PDA from above. (**m**) Colonies on PDA from below. Scale bars: (**d**) 100 µm; (**e**–**k**) 10 µm.

*Endocalyx ptychospermatis* Y.R. Xiong, Manawas & K.D. Hyde, *Fungal Diversity* 114: 327–386 (2022) (Figure 7). 

*Saprobic* on decaying branches of *Trachycarpus fortunei* (Palmae). Sexual morph: Undetermined. Asexual morph: *Conidiomata* 600–640 × 300–379 µm ($\bar{x}$ = 625 × 350 µm, *n* = 10), raised, cup-shaped or cylindrical, scattered or aggregated, pale yellow to pale green, surrounded by numerous yellow hyphal rings. *Conidiophores* 2–2.5 µm ($\bar{x}$ = 2.4 µm, *n* = 10) wide, filiform, hyaline, septate. *Conidiogenous cells* hyaline, integrated, globose or cylindrical. *Conidia* 11.5–16 × 10.5–13.5 µm ($\bar{x}$ = 14 × 12 µm, *n* = 30), unicellular, elliptical to irregular polygonal, pale brown when immature, dark brown to blackish brown when aged, some with a longitudinal germ slit, rough-walled.

Culture characteristics: Conidia germinating on PDA within 12 h at 25 °C. Colonies growing on PDA reaching 1.2–1.4 cm diam. after one week at 25 °C in the dark; white, undulate at the edge, rough from above; dull white from below.

Material examined: China, Sichuan Province, Chengdu city, Pidu district, 30°45′25.01″ N, 103°55′21.97″ E, elevation 466 m, on dead branches of *Trachycarpus fortunei* (Palmae), 30 November 2020, W.L. Li, W71a (HUSET 23.0129), living culture UESTCC 23.0129; W71b (HUEST 23.0130), living culture UESTCC 23.0130. 

Notes: Our collection is similar to *Endocalyx ptychospermatis* in terms of the shape and size of conidiomata and conidia. They were collected from different species of palm (*Trachycarpus fortune* vs. *Ptychosperma macarthurii*) [15]. The phylogenetic analyses indicated that the new collection clustered with the type of *E. ptychosepermatis* (ZHKUCC 21-0008) with 100% ML/1.00 PP statistical support (Figure 2).

## 4. Discussion

Xylariomycetidae is a taxonomically complex fungal group, and most of its taxa were classified based on their morphological characteristics before the wide application of molecular data [50,51,52]. However, phylogenetic studies have indicated that fungi with similar spore-bearing structures have not evolved from the same ancestral lineages [53].

Stromatic characteristics, such as the length of the ascus stipe [51], amyloid reactions [54], and the shape and size of the ascus apical ring [55], are used to delimit xylarialean taxa. The number of ascospores per ascus [56], color [57], septation [23], appendage [54,58] and character of the germ slit [1,12] also play a vital role in the identification of xylarialean taxa. Though morphological characteristics are used for traditional generic circumscriptions (such as stromal morphology), they do not commonly reflect phylogenetic relationships. Several anthostomella-like genera (e.g., *Anthostomella*, *Apiospora*, *Entosordaria*, *Occultitheca*, *Pyriformiascoma* and *Vamsapriya*), with dwarf cells of ascospores, were shown to be morphologically comparable but are phylogenetically distinct throughout Xylariomycetidae [1]. Therefore, the morphology of dwarf cells does not reflect phylogenetic relationships at the familial level but could be informative at the generic level. The new genus *Bicellulospora* has inequilateral ascospores with an olivaceous brown dwarf cell, distinguishing it from other similar genera such as *Entosordaria* and *Castellaniomyces rosae* (Xylariales genera *incertae sedis*), as they differ in morphology. Based on the molecular data and morphological comparison, a new monotypic genus, *Bicellulospora*, with type species *B. elaeidis*, is hereby established and placed in Xylariales as genera *incertae sedis.*

Samarakoon et al. [23] considered that the number of septa cannot be used as a basis for classification at the genus level. Consequently, *Lepteutypa* was synonymized to *Amphisphaeria* based on its holomorphic morphology and multi-gene phylogeny. In our study, two new species (*A. oleae* and *A. verniciae*) and *A. micheliae* were isolated from woody oil plants in Sichuan Province, China. *Amphisphaeria oleae* and *A. micheliae* share one-septate ascospores, while *A. camelliae* and *A. verniciae* clustered close to *A. curvaticonidia*, as they all have three-septate ascospores. Though the number of septa has less taxonomic significance for generic delimitation, it can properly reflect interspecific relationships. 

*Endocalyx* was introduced by Berkeley and Broome [59] and is characterized by sporodochial or synnematous, cylindrical to cup-shaped conidiomata enclosed by yellow or brown sterile peridial hyphae; hyaline to subhyaline, basauxic conidiophores bearing unicellular, elliptical and brown conidia, with a smooth or echinulate surface; and a hyaline germ slit. Delgado et al. [14] conducted a comprehensive assessment of *Endocalyx* based on the molecular data from specimens and strains collected in Japan, Hawaii and the continental U.S.A. Four species were eventually accepted in *Endocalyx* (*E. cinctus*, *E. indumentum*, *E. grossus* and *E. melanoxanthus*). Recently, a fifth species, *E. ptychospermatis*, was reported after its identification in a dead petiole of *Ptychosperma macarthurii* (palm) in China [15]. Here, we provide another collection of *E. ptychospermatis* from *Trachycarpus fortunei* (Palmae) as a new host record.

## Figures and Tables

**Table 1:** Comparison of Bicellulospora and Anthostomella species.

Species	Clypeus	Apical Ring		Ascospores			Germ Slit	Sheath	References
		Shape	Size (μm)	Color of Dwarf Cells	Size of Dwarf Cells (μm)	Larger Cell			
*Anthostomella castnopsis*	Absent	Wedge-shaped	5–8 × 5–6.5	Hyaline	5–8 × 5–6.5	Hyaline	Absent	Absent	[49]
*A. clypeata*	Not dense, black or sometimes with a halo around the central pore, subglobose	Wedge-shaped	/	Hyaline	2–3 × 2–2.5	Brown	Longitudinal, very fine	Absent	[47]
*A. cynaroides*	Black, ellipsoidal, margin indistinct	Wedge-shaped	4.5–5 × 3.5–4	Hyaline	1.5–2 × 3	Brown	Absent	Absent	[47]
*A. formosa*	Absent	Absent	/	Hyaline	1.5–2	Dark brown	Longitudinal	Narrow hyaline	[45]
*A. proteae*	Absent	Wedge-shaped	4–5 × (2–)3	Hyaline	1.5–2 × 2–3	Dark brown	Longitudinal, indistinct	Gelatinous	[47]
*A. tomicoides*	Dense, with conspicuous blackened patches	/	2 × 3	Hyaline	1.5–2	Brown	Longitudinal, indistinct	Present	[46]
*Bicellulospora elaeidis*	Black, subglobose	Discoid	0.8–1.6 × 1.4–2	Hyaline, olivaceous green	3.3–4.4 × 3.3–4.2 µm	Hyaline, yellow, dark brown	Absent	Absent	This study

## Data Availability

All sequence data are available in NCBI GenBank following the accession numbers in the manuscript.

## Supplementary Materials

The following supporting information can be downloaded at: https://www.mdpi.com/article/10.3390/jof10030189/s1, Table S1: Taxa used in this study and their GenBank accession numbers.
